# Changes in the 6th edition of the World Health Organization classification of tumours of the digestive system

**DOI:** 10.1111/his.70116

**Published:** 2026-02-22

**Authors:** Mark J Arends, Irene Esposito, Anthony J Gill, Ralph H Hruban, Joseph D Khoury, Motohiro Kojima, Elizabeth A Montgomery, Fatimah Abdulkareem, Fátima Carneiro, Guido Costamagna, Gregory Lauwers, Alexandros D Polydorides, Guido Rindi, Massimo Rugge, Peter Schirmacher, Amitabh Srivastava, James Yao, Jennelle C Hodge, James G Kench, Bharat Rekhi, Miguel Reyes‐Múgica, Antonia R Sepulveda, Chanjuan Shi, Pavitratha Puspanathan, Harshima Wijesinghe, Christine Giesen, Blanca Iciar Indave Ruiz, Dilani Lokuhetty, WCT standing editorial board, Wendy Cooper, Michael Eden, Andrew Field, Vicky Goh, Jennelle Hodge, James Kench, Joseph Khoury, Katia Leite, Zhiyong Liang, Daichi Maeda, George Netto, Bharat Rekhi, Miguel Reyes Múgica, Brian Rous, Ales Ryska, Shahin Sayed, Antonia R Sepulveda, Chanjuan Shi, Gary Tse, Iris D Nagtegaal

**Affiliations:** ^1^ Edinburgh Pathology, Institute of Genetics & Cancer University of Edinburgh Edinburgh UK; ^2^ Institute of Pathology Heinrich Heine University and University Hospital of Dusseldorf Düsseldorf Germany; ^3^ NSW Health Pathology, Department of Anatomical Pathology Royal North Shore Hospital St Leonards Sydney New South Wales Australia; ^4^ Sydney Medical School University of Sydney Sydney New South Wales Australia; ^5^ Departments of Pathology and Oncology, The Sol Goldman Pancreatic Cancer Research Center The Johns Hopkins University School of Medicine Baltimore Maryland USA; ^6^ Department of Pathology, Microbiology, and Immunology University of Nebraska Medical Center Omaha Nebraska USA; ^7^ Department of Surgical Pathology Kyoto Prefectural University of Medicine Graduate School of Medical Science Kyoto Japan; ^8^ Department of Pathology University of Miami Miller School of Medicine Miami Beach Florida USA; ^9^ Department of Anatomic & Molecular Pathology College of Medicine, University of Lagos Lagos Nigeria; ^10^ Ipatimup/i3S, Faculdade de Medicina da Universidade do Porto and Unidade Local de Saúde São João Porto Portugal; ^11^ Centre of Excellence of Gastrointestinal and Endocrine Metabolic Diseases Ospedale Isola Tiberina – Gemelli Rome Italy; ^12^ Department of Pathology H. Lee Moffitt Cancer Center Tampa Florida USA; ^13^ Department of Pathology, Molecular, and Cell‐Based Medicine Icahn School of Medicine at Mount Sinai New York New York USA; ^14^ Section of Anatomic Pathology, Department of Life Sciences and Public Health Università Cattolica del Sacro Cuore ‐ Unit of Anatomic Pathology, Department of Laboratory and Hematological Sciences, Fondazione Policlinico Universitario Agostino Gemelli IRCCS Rome Italy; ^15^ Pathology and Cytopathology Unit University of Padova Padova Italy; ^16^ Institute of Pathology University Hospital, Heidelberg University Heidelberg Germany; ^17^ Department of Pathology and Laboratory Medicine Memorial Sloan Kettering Cancer Center New York New York USA; ^18^ Gastrointestinal Medical Oncology University of Texas M. D. Anderson Cancer Center Houston Texas USA; ^19^ Department of Medical and Molecular Genetics, and IU Simon Comprehensive Cancer Center Indiana University Indianapolis Indiana USA; ^20^ Department of Tissue Pathology and Diagnostic Oncology Royal Prince Alfred Hospital, NSW Health Pathology Camperdown New South Wales Australia; ^21^ Department of Pathology Tata Memorial Centre Mumbai India; ^22^ Department of Pathology and Laboratory Medicine University of Miami Miller School of Medicine Miami Florida USA; ^23^ Department of Pathology George Washington University, School of Medicine and Health Sciences Washington DC USA; ^24^ Department of Pathology Duke University Medical Center Durham North Carolina USA; ^25^ International Agency for Research on Cancer, World Health Organisation Lyon France; ^26^ Department of Pathology Radboudumc Nijmegen the Netherlands

**Keywords:** biliary system, classification, digestive system, gastrointestinal, liver, neoplasm, pancreas, tumour

## Abstract

The 6th Edition of the WHO Classification of Digestive System Tumours represents a significant update to the 5th edition. It integrates pathological, new molecular, and clinical insights to refine the taxonomy of digestive system neoplasms. The revised classification continues to emphasise standardisation in terminology, coding, and diagnostic criteria to facilitate global consistency in diagnosis, treatment, epidemiological reporting and research. Structural reorganisation of book chapters describes epithelial tumours by anatomical site, while separating neuroendocrine, mesenchymal and haematolymphoid tumours into dedicated chapters that are aligned with other WHO tumour volumes. Genetic tumour syndromes are classified by mechanisms, pathways and genes, whereas metastatic disease is comprehensively covered under other tumours and metastases. Key structural and diagnostic refinements include consolidation of gastric dysplasia entities; separation of duodenal/ampullary from jejuno‐ileal tumours; clearer categorisation of colorectal serrated polyps and novel carcinoma grading; introduction of small‐ and large‐duct intrahepatic cholangiocarcinoma as separate entities, and redefinition of undifferentiated carcinoma to include ‘carcinoma with mesenchymal differentiation’. Several new entities are introduced, including oesophageal epidermoid metaplasia, colorectal intramucosal adenocarcinoma, low‐grade tubuloglandular adenocarcinoma and lymphoglandular complex‐like adenocarcinoma, intraductal tubulopapillary and intraductal oncocytic papillary neoplasms of the bile ducts and sonic hedgehog hepatocellular adenoma. The concept of amphicrine‐like carcinoma (ALC) is distinguished from MiNEN and broadens the understanding of tumours with dual neuroendocrine–non‐neuroendocrine differentiation. Grading systems are simplified to two‐tier classifications (low/high grade) across precursor lesions, with enhanced criteria for neuroendocrine tumour grading. Anal canal neoplasia terminology is harmonised with human papillomavirus (HPV) related Lower Anogenital Squamous Terminology (LAST) and mass‐forming biliary and gallbladder cancer precursors share similar terminology. Finally, carcinoma of unknown primary (CUP) is included in a separate section for the first time, classified by molecular and immunophenotypic profiles to guide therapy. Overall, the 6th edition strengthens tumour diagnostic precision and molecular alignment across the digestive system.

## Graphical abstract

Changes and new entities in the 6th edition WCT of the digestive system, including structural, molecular, and diagnostic updates to standardize terminology and improve clinical relevance. Epithelial tumours are organized by site; neuroendocrine, mesenchymal and haematolymphoid tumours have dedicated chapters, with refined grading, redefined undifferentiated carcinoma, and unified approaches to dysplasia/precursors.

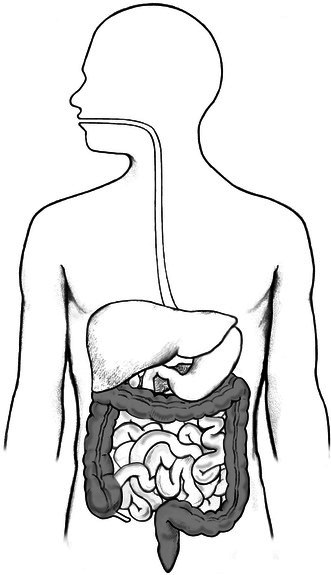

**Abbreviations**
ALC: amphicrine‐like carcinoma
AMN: appendiceal mucinous neoplasm
CUP: carcinoma of unknown primary
ECL cell: enterochromaffin‐like cell
GIST: gastrointestinal stromal tumour
HPV: human papillomavirus
IAPN: intra‐ampullary papillary‐tubular neoplasm
IBD: inflammatory bowel disease
ICN: intracholecystic neoplasm
ICPN: intracholecystic papillary neoplasm
IPNB: intraductal papillary neoplasms of bile duct
ISSVA: International Society for the Study of Vascular Anomalies
ITPN: intraductal tubulopapillary neoplasm
LAST: Lower Anogenital Squamous Terminology
MCN: mucinous cystic neoplasm
MCP: mucinous carcinoma peritonei
MEN1: multiple endocrine neoplasia 1
NEC: neuroendocrine carcinoma
NET: neuroendocrine tumour
OGA: oxyntic gland adenoma
PPI: proton pump inhibitor
SDH: succinate dehydrogenase
SHH‐HCA: sonic hedgehog hepatocellular adenoma
SIL: squamous intraepithelial lesion
SSL: sessile serrated lesion
TSA: traditional serrated adenoma
WCT: Classification of Tumours
WHO: World Health Organization

## Introduction

The classification of digestive system tumours has evolved over many editions of the World Health Organization (WHO) Classification of Tumours (WCT), guided by advancements in our understanding of the morphology, pathophysiology and molecular pathology characteristics of these tumours. Digestive system tumours arise across different sites and from various tissues within the digestive system, each exhibiting distinct gross and microscopic pathologies, biological behaviours and clinical outcomes. The WCT has played a pivotal role in standardising the classification of digestive system tumours, providing a comprehensive framework that categorises these neoplasms based on the anatomical location, histological features, molecular characteristics and clinical implications. This classification system not only aids in the accurate diagnosis and treatment of digestive system tumours but also facilitates epidemiological studies and research by enabling consistent data collection and comparison across studies. Throughout the volume, the focus is on offering unequivocal and universally acceptable terminology for neoplastic diseases,^1^, ^2^ which is essential as the basis for future research (including artificial intelligence and molecular pathology studies) and evidence‐based treatment.^3^, ^4^ Sensitive to the challenges of molecular testing in low‐income countries, molecular testing is employed in tumour classification only when necessary. This volume also provides ICD‐O 4 coding with 5‐digit morphology and updated topography codes and updated epidemiology, clinical features and pathological staging.

In this review, we focus on structural and re‐organisational changes, new entities and selected improvements compared with the previous edition. We also describe the different systematic approaches applied to this new edition of WCT of the digestive system.

## Structural changes and reorganisation

The larger part of the volume reflects the high frequency of epithelial neoplasms of the digestive system. Both premalignant and malignant epithelial neoplasms are categorised by their primary anatomical site, arranged along the cranial to the caudal axis. There are separate chapters for neuroendocrine neoplasms, as well as for the non‐epithelial tumours, including haematolymphoid and mesenchymal tumours. These chapters have been developed in close collaboration with the current WCT volumes for these sets of tumours, ensuring consistent terminology, definitions and updated information.

The approach to haematolymphoid tumours utilised the 5th edition WHO Classification of Haematolymphoid Tumours^5^ as the primary source, from which tumour types were selected based on the following criteria: (1) entities that arise specifically in the digestive system; (2) entities with a particular tropism for the digestive system and (3) entities that involve the digestive system with relatively high frequency. For mesenchymal tumours, a similar approach was chosen, with a more general overview of soft tissue pathology in the introduction to cover those entities that are rare or extremely rare in the digestive system. Mesenchymal tumours with similar features across body sites, such as solitary fibrous tumour, are featured in the introduction rather than as separate sections and are described in greater detail in the WHO Classification of Soft Tissue and Bone Tumours.^6^ Gastrointestinal stromal tumour (GIST) is retained in the mesenchymal tumours chapter with succinate dehydrogenase (SDH)‐deficient GIST as a separate section. Harmonisation is ensured for mesenchymal tumours between the 6th edition soft tissue tumours classification and the corresponding tumours in the digestive system, especially in terms of the definition and content, as much as possible. Another update included are the sections corresponding to vascular neoplasms and other vascular anomalies affecting the digestive system. We have included the vascular neoplasm updates described in the 5th edition of the WHO Classification of Paediatric Tumours,^7^ incorporating the current International Society for the Study of Vascular Anomalies (ISSVA) classification system,^8^, ^9^ harmonising nomenclature across other WHO volumes published and in preparation. In contrast to the 5th edition, where the neuroendocrine neoplasms were described in each of the organ‐specific chapters, these neoplasms are now gathered into a separate chapter, allowing greater alignment of neuroendocrine tumour features throughout the digestive system and with those in other organ systems set out in the 5th edition volumes. The chapter covering the genetic tumour syndromes is similarly aligned with the 5th edition WHO Classification of Genetic Tumour Syndromes^10^ based on major mechanism, pathway, syndrome and affected gene(s), with updating and some additions. As in the 5th edition, those tumours that did not fit into the above‐mentioned categories were assembled in an ‘other tumours’ chapter, which includes a more extensive description of metastatic disease as well. In addition, within the various chapters, several sections were reorganised, as summarised in Table 1.

**Table 1:** Reorganisation of the classification of digestive system tumour entities in the 6th edition in comparison with the 5th edition of the digestive system tumour classification

Chapter	Tumour type	Subject
Oesophagus	Carcinomas with combined squamoid and glandular components	Adenosquamous carcinoma, mucoepidermoid carcinoma and adenoid cystic carcinoma
Stomach	Gastric tumour precursors	Gastric dysplasia, intestinal‐type adenomas and gastric‐type adenomas
	Gastroblastoma	Moved to mesenchymal chapter
Small bowel	All tumours	Split into those of the duodenum and ampulla, and those of the jejunum and ileum
Colorectum	Serrated polyps	Split into hyperplastic polyps, sessile serrated lesions and traditional serrated adenomas
Other tumours and metastases	Digestive system metastases are separated into three sections	Split into liver metastases, peritoneal metastases and metastases to other digestive system sites (in addition to carcinoma of unknown primary, CUP)
	Peritoneal metastases subdivided	Split into metastatic nonappendiceal tumours and metastatic appendiceal mucinous neoplasms
Genetic tumour syndromes	Serrated polyposis	Acknowledgment of mostly nongermline origin, moved to the colorectal chapter

### Oesophagus

In recognition of their mixed morphology, with both squamoid and glandular components, organisational refinements were made to group together adenosquamous carcinoma, mucoepidermoid carcinoma and adenoid cystic carcinoma to allow comparison and distinction between these entities that share mixed features. Mucoepidermoid carcinoma, adenoid cystic carcinoma and adenosquamous carcinoma are all rare (<1% of primary oesophageal carcinomas) and mostly arise in men (median age about 60 years).^11^, ^12^, ^13^, ^14^, ^15^, ^16^, ^17^ Oesophageal mucoepidermoid carcinoma and oesophageal adenoid cystic carcinoma may arise from oesophageal submucosal glands.^11^, ^18^ Adenosquamous carcinoma likely derives from either a primary squamous cell carcinoma or primary adenocarcinoma associated with Barrett oesophagus, manifesting divergent glandular and squamous differentiation from a common precursor, a concept supported by shared mutation profiles.^19^, ^20^ Mucoepidermoid and adenoid cystic carcinomas recapitulate their salivary gland counterparts. Mucoepidermoid carcinoma shows variable fractions of cysts and sheets of mucin‐producing epithelial cells, epidermoid cells and intermediate cells, and characteristically harbours *MAML2* gene rearrangements. Adenoid cystic carcinoma consists of epithelial and myoepithelial cells in a bilayer with inner epithelial and outer myoepithelial cells, organised in a variety of cribriform, tubular (glandular), or solid patterns. It often shows punched‐out spaces filled with hyaline material. Detecting *MYB* gene rearrangements and/or overexpression of MYB protein on immunohistochemistry can confirm the diagnosis.^21^, ^22^, ^23^ Adenosquamous carcinoma shows distinct components of adenocarcinoma and squamous cell carcinoma that are separate or intermingled with each other. The volume of each component has not been shown to be relevant, but an arbitrary requirement for at least 20% of each of them has been suggested; otherwise, defaulting to the majority component for classification.^24^

### Stomach

A key modification in the gastric chapter is the approach to dysplasia. Elevated gastric dysplastic lesions have been traditionally classified as adenomas, but they develop in a similar way to visible colorectal colitis‐associated dysplasia. In contrast to sporadic adenomas, both of these arise in damaged mucosa. For this reason, the classification is modified in this edition to mirror the approach to management of epithelial precancerous conditions and lesions of the stomach (MAPS III) guidelines.^25^, ^26^, ^27^ Sections previously dedicated to gastric dysplasia, intestinal‐type adenomas, and gastric‐type adenomas are now combined into one section under gastric dysplasia. Special care has also been taken to address the recently recognised gastric dysplastic lesion that develops in *Helicobacter pylori‐*naive stomachs and presents with a foveolar phenotype.^28^, ^29^, ^30^ Lesions arising beneath the gastric foveolar surface, including both oxyntic gland neoplasms and pyloric gland adenomas, are separated.

In several case series, gastroblastoma is recognised as the most prevalent low‐grade biphasic epithelial‐mesenchymal neoplasm, characterised by *GLI1* fusions.^31^ These GLI1‐altered neoplasms most commonly arise in the distal stomach. However, since examples have been reported in the proximal stomach, small bowel (‘enteroblastoma/GLI1‐rearranged enteric tumour’),^31^ oesophagus, and liver,^32^, ^33^, ^34^, ^35^, ^36^, ^37^ as well as in numerous other sites throughout the body, they are all now subsumed under the umbrella term ‘GLI1‐altered neoplasms’. Gastroblastomas are usually biphasic, composed of variable proportions of monotonous spindle cells and uniform epithelial cells arranged in nests, trabeculae and pseudorosettes, although rare monophasic epithelial cases have been noted.^36^ The spindle cell component consists of slender tumour cells in sheets and bundles set in an often‐myxoid and occasionally calcified stroma. The epithelial component consists of bland cells with moderate amounts of pale eosinophilic cytoplasm and ovoid nuclei with open chromatin. This entity is transferred to the mesenchymal chapter to reflect the key importance of the mesenchymal component.

### Small bowel

Based on differences in epidemiological, anatomical, and biological features, the 6th edition splits tumours of the small bowel into those of the duodenum and ampulla and those of the jejunum and ileum. The duodenum is the most common site for small intestinal neoplasms,^38^, ^39^ with the ampulla being the most common localisation within the duodenum, followed by the descending duodenum.^40^, ^41^ This approach also recognises that duodenal and ampullary neoplasms have overlapping surgical treatments. Mass‐forming ampullary precursor lesions are classified under the term ‘Ampullary adenomatous neoplasms’ according to careful topographic, gross and histopathology features, and the anatomic classification of tumours of the ampulla was refined further (described later).

Tumours in the jejunum and the ileum share many features with their colorectal counterparts, although there is considerably less knowledge about small bowel tumours. They are much less frequent and less accessible to endoscopy for biopsy or endoscopic resection, thus reducing the number of early diagnostic samples available for research. Small bowel malignancies with the highest incidence are metastatic tumours, covered under the chapter on ‘Other tumours and metastases’.

### Large bowel

In the colorectal tumours chapter, increased recognition of serrated polyps led to the subdivision of these into three types: hyperplastic polyps, sessile serrated lesions (SSL) and traditional serrated adenomas (TSA). Thus, the spectrum of conventional and serrated precursors is now better defined and illustrated. Reflecting the larger case load of colorectal polyps due to population bowel screening programmes, more extensive descriptions of hamartomatous juvenile polyps (formerly only described as part of juvenile polyposis syndromes) are included. Similarly, Peutz–Jeghers polyps are described in the chapter on tumours of the jejunum and ileum.

### Serrated polyposis

The section on colorectal serrated polyposis has been moved from the chapter of genetic tumour syndromes to the colorectal chapter, since germline (constitutional) pathogenic variants in *RNF43* have only been reported in around 2% of patients^42^, ^43^, ^44^ and no other high‐penetrance candidate genes have been identified over the past 2–3 decades. Serrated polyposis or multiple serrated polyps may present as part of one of the other polyposis syndromes, including PTEN hamartomatous tumour syndrome and hereditary mixed polyposis syndrome.^45^, ^46^, ^47^

### Appendix

In the 6th edition, appendiceal epithelial neoplasms are classified as benign and malignant, with the former category including serrated lesions as well as new, separate entries for conventional appendiceal adenoma, which resembles its colorectal counterpart, and mucinous adenoma, a diagnostic term that is reinstated in this edition. Mucinous adenoma consists of circumferential mucinous neoplastic epithelium confined to the mucosa and completely surrounded by an intact muscularis mucosae. Not clearly defined in the prior edition and vaguely grouped with appendiceal mucinous neoplasms (AMNs), this distinction reflects our current understanding that these are benign lesions, cured by appendectomy.^48^ In contrast, AMNs are now classified under malignant epithelial neoplasms, together with adenocarcinomas and goblet cell adenocarcinomas, reflecting their potential for spread in some cases, particularly in the form of peritoneal mucinous ascites (pseudomyxoma peritonei). The latter, together with its histological counterpart termed mucinous carcinoma peritonei (MCP), are now separately discussed under the peritoneal metastases section.

### Metastases

Metastases to the digestive system are described in the ‘other tumours and metastases’ chapter. Currently, this is covered by four sections: liver metastases, peritoneal metastases, metastases to other digestive system sites, and carcinoma of unknown primary (CUP). The peritoneal metastasis section is further subdivided into metastatic non‐appendiceal tumours and metastatic AMNs. This subdivision acknowledges that, although appendiceal tumours are a relatively uncommon cause of peritoneal metastasis, AMNs are the most frequent cause of the distinctive clinical syndrome of pseudomyxoma peritonei.^49^, ^50^, ^51^ In addition to peritoneal metastasis from low‐grade and high‐grade AMNs (LAMN and HAMN), this section covers peritoneal metastases from appendiceal adenocarcinomas and discusses the differential diagnosis with other entities.^52^

## New entities

**Figure 1:**
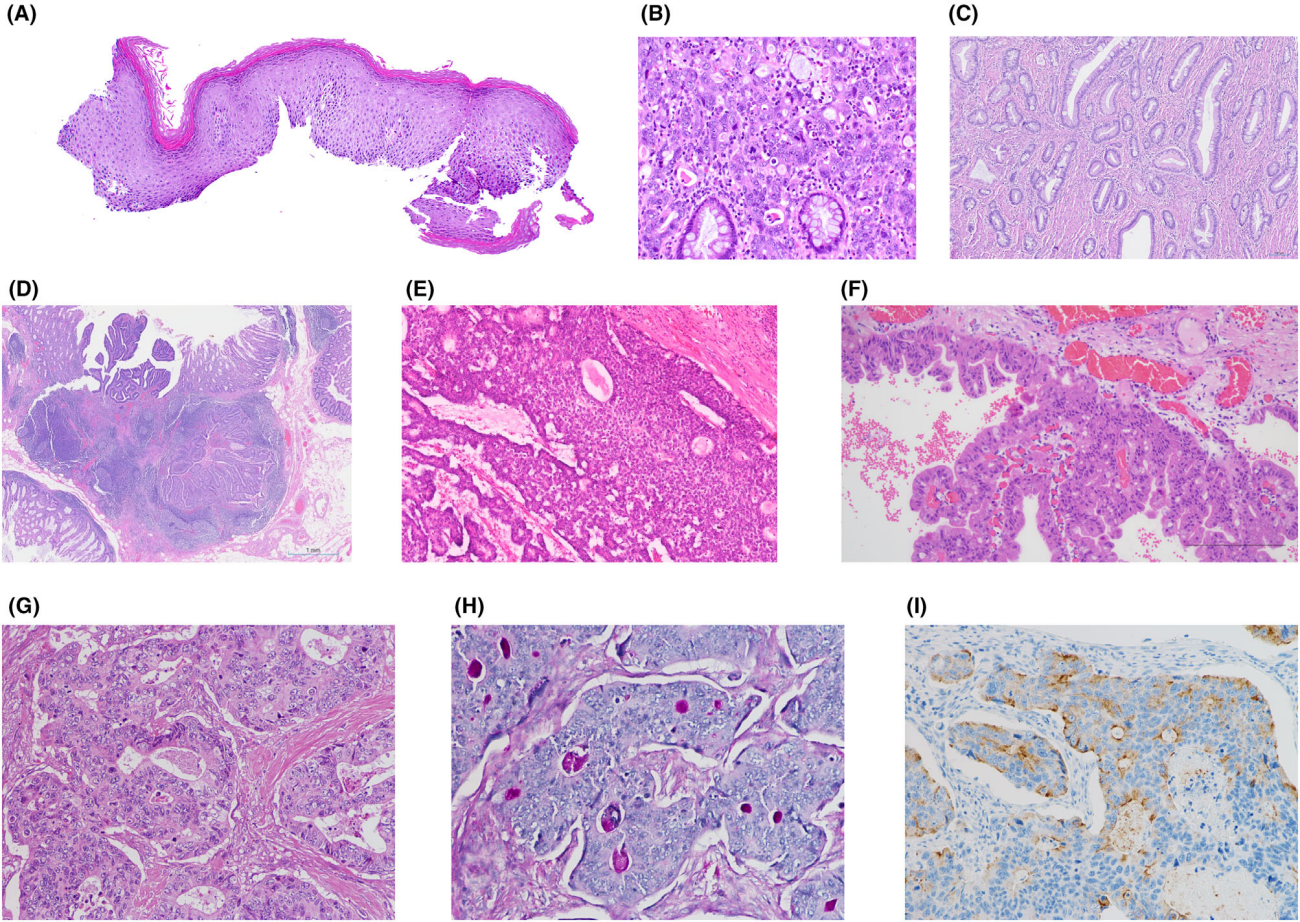
New entities. (**A**) Oesophageal epidermoid metaplasia, (**B**) colorectal intramucosal carcinoma, (**C**) colorectal low‐grade tubuloglandular adenocarcinoma, (**D**) colorectal lymphoglandular complex‐like adenocarcinoma, (**E**) intraductal tubulopapillary neoplasm (ITPN) of the bile duct, (**F**) intraductal oncocytic papillary neoplasm (IOPN) of the bile duct, (**G**) amphicrine‐like carcinoma (ALC) (H&E), (**H**) amphicrine‐like carcinoma showing mucin production (DiPAS), and (**I**) amphicrine‐like carcinoma displaying evidence of neuroendocrine differentiation (chromogranin A immunohistochemistry).

The 6th edition of the WCT of the digestive system reflects the increasing knowledge of digestive system neoplastic entities, with improved refinement mostly due to novel molecular insights. Several new entities have been recognised since the last edition and are described in the relevant chapters. Recognition of a new entity or (sub)type requires them to be different from the main tumour type in at least two, but often more, significant characteristics (clinical, histological, or molecular), preferably resulting in a different treatment or outcome. The new entities in this 6th edition are summarised in Table 2 and most are illustrated in Figure 1.

**Table 2:** New neoplastic entities of the digestive system tumour classification (6th edition)

Tumour type	Definition	Relevance
*Oesophagus*		
Oesophageal epidermoid metaplasia	Epidermoid metaplasia of the oesophagus is a sharply demarcated area of epithelial hyperplasia with a prominent granular cell layer and hyperorthokeratosis	Precursor to squamous cell carcinoma
*Colon and rectum*		
Intramucosal carcinoma	Unequivocal infiltration in the lamina propria	Identification of patients who are at high risk for additional colorectal carcinoma (CRC)
Low‐grade tubuloglandular carcinoma	Infiltration by haphazard, well‐differentiated glands without desmoplasia	Inflammatory bowel disease (IBD)‐associated distinctive morphological subtype is difficult to diagnose as invasive
Lymphoglandular complex‐like adenocarcinoma	Lymphoglandular complexes with desmoplasia and necrosis	Distinctive CRC subtype, present at an early stage, seen in screening programmes
*Liver*		
Sonic hedgehog‐related hepatocellular adenoma (shh‐HCA)	Small somatic deletions that fuse the promoter of *INHBE* with *GLI1*	Distinctive neoplasm subtype with a high risk of haemorrhage
*Bile ducts*		
Intraductal tubulopapillary neoplasm (ITPN)	Noninvasive, intraluminally growing epithelial neoplasm without significant mucin production, primarily composed of high‐grade dysplastic epithelial cells that form back‐to‐back tubules and, less frequently, papillary structures	Distinctive neoplasm subtype with high risk of progression to carcinoma, but relatively good survival
Intraductal oncocytic papillary neoplasm (IOPN)	Noninvasive intraductal epithelial tumour characterised by exophytic nodular projections, composed of epithelium with abundant eosinophilic cytoplasm	A distinctive neoplasm subtype that is relatively rare, with an intermediate outcome once it progresses to carcinoma
*Pancreas*		
Simple mucinous cyst	Non‐invasive cavity‐forming lesion (>1.0 cm) composed of gastric‐type epithelium with an absence of papillary architecture and ovarian‐type stroma	A distinctive neoplasm subtype, in the absence of dysplasia/carcinoma, is associated with a good outcome
*Mesenchymal*		
Succinate dehydrogenase (SDH)‐deficient gastrointestinal stromal tumour (GIST)	Mesenchymal neoplasm that shows differentiation towards the interstitial cells of Cajal and is driven by dysfunction of the mitochondrial complex 2 (known as *succinate dehydrogenase deficiency*)	Distinctive neoplasm subtype, strongly syndromic
NUT‐rearranged sarcoma	Aggressive neoplasm characterised by NUT rearrangement	Recently identified distinctive neoplasm subtype with aggressive behaviour
*Neuroendocrine*		
Type 4 ATP4A mutation‐associated enterochromaffin‐like cell (ECL‐cell) neuroendocrine tumour (NET)	ECL‐cell gastric NET seen in patients with an inactivating mutation of ATP4A	Distinctive neoplasm subtype associated with severe hypergastrinemia
Type 5 proton pump inhibitor‐related ECL‐cell NET	ECL‐cell gastric NET seen in patients on long‐term proton pump inhibitor use	Distinctive neoplasm subtype associated with moderate hypergastrinemia
Amphicrine‐like carcinoma	‘Amphicrine’ indicates dual exocrine and neuroendocrine differentiation, either in the same tumour cells, or in different tumour cells that are intimately admixed	Amphicrine‐like carcinoma (ALC) should be staged according to the relevant TNM systems applicable to its non‐neuroendocrine carcinoma counterparts

### Oesophagus

Oesophageal epidermoid metaplasia is a new entry in the 6th edition (Figure 1A). It is postulated that epidermoid metaplasia is due to chronic oesophageal injury.^53^ Recurrent damage to the oesophageal mucosa leads to its transformation into a keratinised, skin‐like squamous epithelium, as a mechanism of cellular adaptation to chronic stress and repeated injury. This epithelial condition has been regularly detected in association with oesophageal squamous neoplasia and in patients at risk for development thereof.^54^, ^55^, ^56^ Furthermore, identical molecular alterations such as somatic *TP53* mutations have been found concurrently in both epidermoid metaplasia^57^ and associated squamous cell carcinoma, consistent with a precursor relationship.

### Large bowel

Intramucosal adenocarcinoma is characterised by unequivocal infiltration of tumour cells restricted to the lamina propria. This diagnosis cannot be reliably made on biopsies and should be restricted to polypectomy and resection specimens. Further, this diagnosis should be restricted to cases with overtly poor differentiation, poorly differentiated clusters, signet‐ring carcinoma cells or tumour budding^58^, ^59^ (Figure 1B). Notably, intramucosal carcinomas have a very low incidence (<0.1% of all colorectal carcinomas), but there is a strong correlation with hereditary cancer syndromes.^58^ The risk of lymph node metastases is negligible.^58^, ^59^

The distinctive colorectal low‐grade tubulo‐glandular adenocarcinoma, typically seen in inflammatory bowel disease (IBD), is now included as a new morphological subtype (Figure 1C). It is a rare subtype, which may arise from low‐grade conventional dysplasia or from non‐conventional colitis‐associated dysplasia in IBD.^60^ CK7 expression is sometimes present. Somatic *IDH1* mutations are common.^61^

The tumour previously described as ‘dome carcinoma’ or ‘gut‐associated lymphoid tissue carcinoma’ in the older literature is now included as colorectal lymphoglandular complex‐like adenocarcinoma (Figure 1D). In general, these tumours present at a low stage^62^, ^63^ and are more frequently encountered in population screening programmes.

### Extrahepatic bile ducts and gallbladder

In the extrahepatic bile ducts, non‐invasive, mass‐forming epithelial tumours now include intraductal papillary neoplasms of bile duct (IPNB), intraductal tubulopapillary neoplasms (ITPN) (Figure 1E), and IOPN (Figure 1F), as separate entities.^64^, ^65^ They are described in the extrahepatic bile duct chapter and are only referred to in the liver and intrahepatic bile duct chapter, with the inclusion of mucinous cystic neoplasm (MCN), which is far more frequent in the liver.^66^, ^67^, ^68^ In the gallbladder, mass‐forming intraepithelial neoplasms have been provisionally designated intracholecystic neoplasms (ICNs), which are now further subcategorised parallel to the existing subtypes in the pancreas.

### Liver

HCA is an almost completely morpho‐molecularly subtyped neoplasia that now includes the newly defined subtype sonic hedgehog HCA (SHH‐HCA), in addition to other subtypes *HNF1A*‐inactivated HCA (H‐HCA), inflammatory HCA (IHCA), β‐catenin activated HCA (B‐HCA) and β‐catenin inflammatory HCA (B‐IHCA). SHH‐HCA, composed of bland and mostly small hepatocytes, accounts for 5–10% of HCAs and is characterised by activation of the sonic hedgehog pathway, usually due to small somatic deletions of *INHBE* leading to *INHBE*–*GLI1* fusions.^69^, ^70^ SHH‐HCAs are associated with a high risk of haemorrhage, but do not display a significant risk of malignant transformation.^69^, ^70^, ^71^, ^72^, ^73^

### Pancreas

Acinar cystic transformation is considered to be a non‐neoplastic cystic lesion and, therefore, is no longer included in the WCT. The 6th edition has introduced the simple mucinous cyst as a neoplastic cystic lesion. A simple mucinous cyst is a cavity‐forming lesion (>1 cm) composed of gastric‐type epithelium with an absence of papillary architecture and ovarian‐type stroma. Somatic alterations in some oncogenes and tumour suppressor genes, including *KRAS*, *BRAF, TP53* and *SMAD4*, have been identified in simple mucinous cysts^74^, ^75^, ^76^, ^77^; therefore, they likely represent a precursor for pancreatic ductal adenocarcinoma.

### Mesenchymal neoplasms

Succinate dehydrogenase‐deficient GIST (*SDH‐*deficient GIST) was previously considered as a subtype of GIST, but is now recognised as a distinct entity due to its unique clinical, histological, immunohistochemical and molecular features, most importantly its strongly syndromic nature.^78^, ^79^, ^80^ *NUT*‐rearranged sarcomas, newly described aggressive neoplasms characterised by variable morphologies and defined by rearrangements in the *NUTM1* or *NUTM2* genes, are also recognised as a distinct tumour type in the gastrointestinal tract for the first time.

### Neuroendocrine neoplasms

Two subtypes of gastric enterochromaffin‐like cell (ECL cell) neuroendocrine tumours (NETs) are added to the previously defined subtypes (type 1 chronic atrophic gastritis associated, type 2 multiple endocrine neoplasia 1 (MEN1) associated, and type 3 sporadic tumours). Type 4 ATP4A‐associated ECL‐cell NETs are seen in patients with an inactivating mutation of *ATP4A*, which impairs gastric acid production and consequently increases gastrin production.^81^, ^82^ These patients typically have severe hypergastrinemia, prominent parietal cell hyperplasia, and many gastric NETs. Type 5 gastric NETs occur in patients on long‐term proton pump inhibitor (PPI) use and are associated with hypergastrinemia.^83^, ^84^, ^85^ Type 5 ECL‐cell NETs can be multifocal, with background oxyntic mucosa showing parietal cell hyperplasia and ECL‐cell hyperplasia.

**Figure 2:**
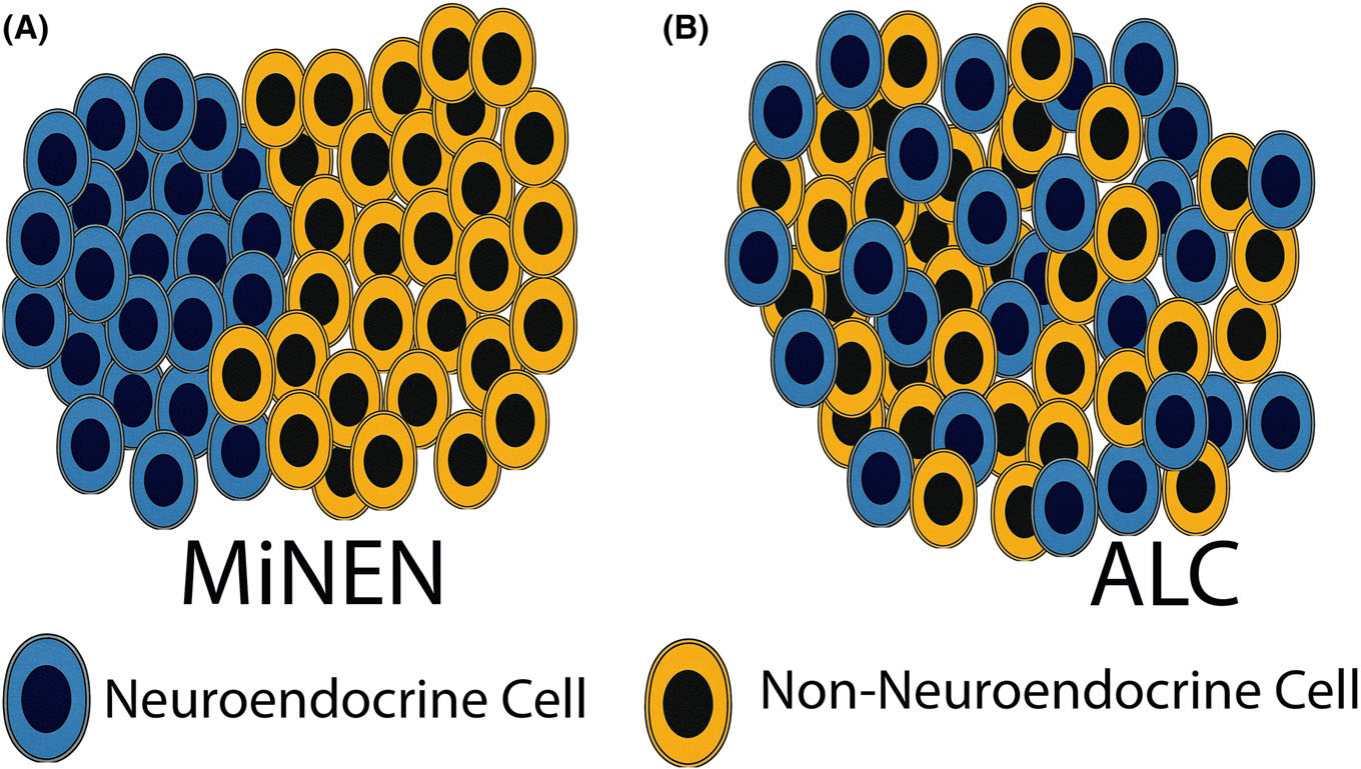
In mixed neuroendocrine and non‐neuroendocrine neoplasms (MiNEN, **A**), the neuroendocrine and non‐neuroendocrine areas form morphologically distinct nodules or zones. In contrast, in amphicrine‐like carcinoma (ALC, **B**), the neuroendocrine and non‐neuroendocrine cells are intimately intermixed throughout the tumour mass.

The concept of amphicrine‐like carcinomas (ALCs) is introduced as a category of neoplasia with mixed neuroendocrine and non‐neuroendocrine differentiation distinct from MiNENs (mixed neuroendocrine–non‐neuroendocrine neoplasms) (Figure 1G–I and Figure 2). In MiNENs, there are morphologically distinct zones or subclonal nodules with either neuroendocrine or non‐neuroendocrine differentiation. In contrast, in ALC the neoplastic cells with different lines of differentiation (neuroendocrine and non‐neuroendocrine) are intimately intermixed, or in some cases, individual cells exhibit both patterns of differentiation (Figure 2). Most commonly, ALCs are composed of morphologically similar appearing cells, some of which express neuroendocrine markers while others express non‐neuroendocrine markers such as mucin. Less common may be co‐expression of both classes of markers in the same cells. Like MiNEN, ALC is not a specific diagnosis but rather a family of tumours. For example, a tumour should not be classified solely as ALC, but rather as amphicrine‐like adenocarcinoma or amphicrine‐like squamous cell carcinoma. Both MiNEN and ALC categories of tumour require morphological and immunohistochemical evidence of neuroendocrine differentiation.

It is recognised that the decision to separate ALCs from other carcinomas may be controversial and their recognition in clinical practice may be difficult. To facilitate practical recognition, detailed guidelines are given in the WHO classification (6th edition)—for example requiring morphological evidence of neuroendocrine differentiation in ≥30% of neoplastic cells with confirmation by immunohistochemistry (requiring at least two neuroendocrine markers to be expressed). The justification for recognising ALCs can be understood by considering the most common and well recognised example—goblet cell adenocarcinoma. Goblet cell adenocarcinoma is the specific diagnosis given to the most common ALC of the vermiform appendix. It demonstrates a significantly worse prognosis than NET but a (slightly) better prognosis than conventional adenocarcinoma of the vermiform appendix. For this reason, goblet cell adenocarcinoma is classified and staged as a carcinoma rather than a NET, but it is important to recognise that the natural history of goblet cell carcinoma is different to both NETs and conventional adenocarcinomas arising in the same organ. The question of whether ALCs of other organs also demonstrate distinctive natural histories can only be answered by the acquisition of further data, which we hope the new classification will facilitate. However, it is already known that the subtype of pancreatic acinar cell carcinoma which is amphicrine‐like behaves similarly to pure acinar cell carcinoma in a way that is intermediate between NET and pancreatic ductal adenocarcinoma, but more similar to adenocarcinoma. It is emphasised that because ALCs are carcinomas they are staged according to the relevant TNM systems applicable to carcinomas and not NETs.

## Selected improvements

The 6th edition classification of tumours of the digestive system permitted the opportunity to improve various features of the neoplastic entities classified to include new developments in knowledge and understanding of digestive system neoplasms. These are improvements and refinements relating to recent novel pathological and molecular research. Although all neoplastic entities are updated in the chapters of the 6th edition, several new improvements or changes of tumour features recognised since the previous edition and selected improvements are highlighted here and summarised in Table 3.

**Table 3:** Selected improvements or changes within the new classification of tumours of the digestive system (6th edition)

Chapter	Tumour type	Subject/topic	Change
General	Undifferentiated carcinoma		Reclassified
Stomach	Oxyntic gland neoplasia		Criteria refined
Duodenum and Ampulla	Ampullary adenomas	Ampullary‐duodenal adenomas and intra‐ampullary papillary‐tubular neoplasms (IAPN)	Separated and distinguished from flat dysplasia (gives origin to ampullary‐duct adenocarcinoma)
Colon and rectum	Larger polyps with combined dysplasia	Clarification of definitions	Classification has been refined to add traditional serrated adenomas with conventional dysplasia
	Colorectal adenocarcinoma	Grading	Improved definition of the determination of high‐grade carcinoma
Anal canal	Anal canal neoplasia terminology	Terminology	Alignment with the Lower Anogenital Squamous Terminology (LAST) project
	Anal intraepithelial neoplasia (AIN)	Molecular characterisation	AIN subclassified: *TP53*‐mutant or *TP53*‐wild type
Liver	Intrahepatic Cholangiocarcinoma	Nomenclature and classification	Intrahepatic cholangiocarcinoma is classified into small‐duct and large‐duct types
Gallbladder and extrahepatic bile ducts	Grading of precursor lesions	Grading	Graded by a two‐tiered classification system
	Mass‐forming precursor lesion	Nomenclature and classification	Intracholecystic neoplasms (ICN), subdivided into intracholecystic papillary neoplasm (ICPN) and intracholecystic tubular neoplasm (ICTN) in keeping with bile‐duct/pancreatic terminology
Pancreas	Pancreatic Neuroendocrine tumour (PanNET)	Grading	Split of grade 2 into grade 2a and grade 2b
Metastases	Carcinoma of unknown primary (CUP)	Subcategorisation	Broadly divided into favourable and unfavourable groups

### General issues: undifferentiated carcinoma

Tumours lacking definite microscopic or immunohistochemical features of squamous, glandular or neuroendocrine differentiation were previously included among ‘undifferentiated carcinomas’ in all gastrointestinal tract sites. For some of these neoplasms, the previously used term ‘carcinoma with sarcomatoid components’ (5th edition) is now replaced by the term ‘carcinoma with mesenchymal differentiation’. Furthermore, the undifferentiated carcinoma category is expanded to include a distinct subset characterised by SWItch/sucrose non‐fermentable (SWI/SNF) chromatin remodelling complex deficiencies, a family that includes somatic alterations in *ARID1A*, *SMARCA2* (*BRM*), *SMARCA4* (*BRG1*) and *SMARCB1* (*INI‐1*), which are all tumour suppressors.^86^, ^87^, ^88^, ^89^, ^90^, ^91^, ^92^, ^93^

### Stomach: oxyntic gland adenoma

Oxyntic gland adenoma (OGA) is now more clearly defined. This small intramucosal lesion consists of cells with mild cytologic atypia, differentiating into predominantly chief cells and, to a lesser extent, parietal cells. A pushing interface with the surrounding mucosa and prolapse‐type misplacement are characteristic of OGA and should not be misinterpreted as evidence of gastric adenocarcinoma of fundic gland type. In biopsy specimens, OGA can be challenging to differentiate from normal oxyntic glands, but the diffuse strong positivity of CyclinD1 in OGA is helpful in separating the two.^94^

### Duodenum and ampulla: carcinomas

**Figure 3:**
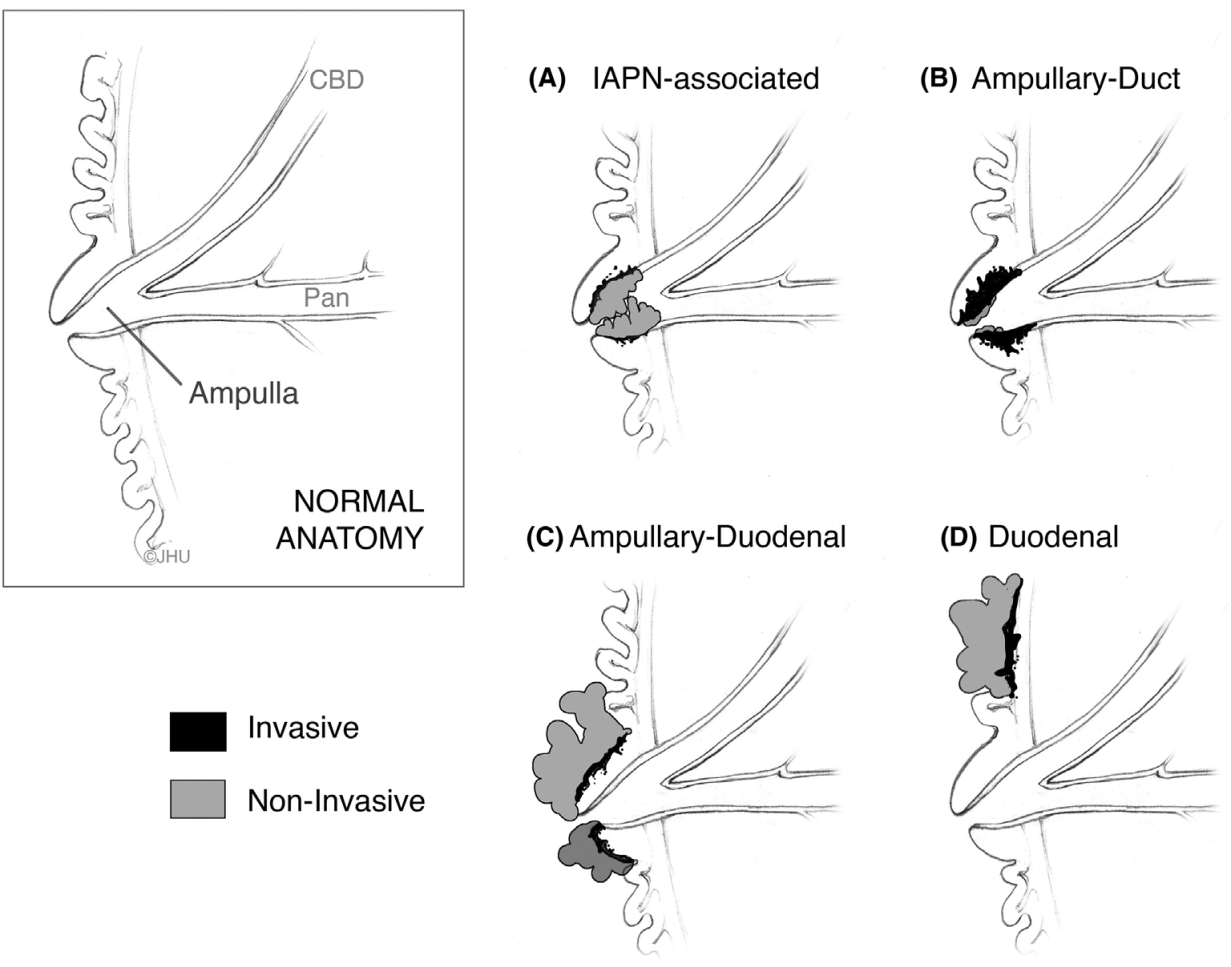
Anatomical types of ampullary region tumours. The four types of adenocarcinomas of the ampullary region include (**A**) intra‐ampullary papillary‐tubular neoplasm (IAPN)‐associated adenocarcinoma, (**B**) ampullary ductal adenocarcinoma (ampullary‐duct); (**C**) ampullary‐duodenal (growing on the duodenal surface of the ampulla); and (**D**) nonampullary duodenal adenocarcinoma (Artwork by Cory Sandone, ©Johns Hopkins University).

The anatomical complexity of the ampulla region drives the subdivision of its adenocarcinomas into four main types of cancer (Figure 3). These include: (1) nonampullary adenocarcinoma (duodenal adenocarcinoma away from the ampulla), (2) ampullary‐duodenal adenocarcinoma, which most commonly presents as an exophytic mass growing on the duodenal surface of the ampulla obstructing the orifice of the papilla, (3) intra‐ampullary papillary‐tubular neoplasm (IAPN)‐associated adenocarcinoma, characterised by intra‐ampullary growth of a papillary precursor lesion coupled with an invasive adenocarcinoma, and (4) ampullary ductal adenocarcinoma that grows in the ampulla and originates from microscopic, mostly flat, high‐grade intraepithelial neoplasia of the ampullary ducts.^95^ The first two entities are often associated with an adenomatous component.^96^ The four main types of duodenal cancers may have different patterns of differentiation and have different prognoses. The prognosis of nonampullary‐duodenal cancer is similar to that of ampullary‐duodenal adenocarcinoma^96^ and of cancers originating from IAPN, which in turn is significantly better than the prognosis of ampullary‐ductal carcinomas arising deep within the ampulla.^97^, ^98^, ^99^, ^100^

### Colorectum: classification of large colorectal polyps

Larger polyps, where conventional dysplasia may be combined with either TSA or SSL, might have been previously diagnosed as mixed polyps. In the 5th edition, SSL with conventional dysplasia was already recognised. In the 6th edition, we added TSA with conventional dysplasia,^101^ since this is now recognised as a distinct pathway of progression for these polyps. An adenomatous polyp with any amount of TSA component should be classified as TSA with low‐grade or high‐grade dysplasia based on nuclear cytology.

### Colorectum: grading of colorectal carcinoma

Grading of colorectal adenocarcinoma, which arguably carries even more clinical importance than histological typing, especially in the context of malignant polyps, is now described with improved clarity. Grading of colorectal adenocarcinomas is based on assessment of gland formation, rather than cytological features, in the least differentiated tumour component. It should be reported as high‐grade when containing at least one focus lacking gland formation in one high‐power field.^102^ Any tumour without such a focus should be reported as low grade. The invasive front, where tumour budding and poorly differentiated clusters may occur as a sign of epithelial–mesenchymal transition, should not be assessed when grading the tumour; though such findings should be reported separately. Cribriform architecture is considered a pattern of glandular differentiation and does not indicate high‐grade morphology. This grading system offers the most prognostic value, especially for microsatellite‐stable tumours.^102^

### Anal canal: terminology

Anal canal neoplasia terminology is now aligned with the terminology of the Lower Anogenital Squamous Terminology (LAST) project,^103^ which reflects an improved understanding of human papillomavirus (HPV) biology and involvement in this neoplasm, thus facilitating clear communication across specialties. As for noninvasive precursor lesions of the anal canal, the term squamous intraepithelial lesion (SIL) has been adopted for harmonisation with genital tract lesions. In SILs, the two‐tiered grading system has been adopted: low‐grade SIL (LSIL) and high‐grade SIL (HSIL) for HPV‐ related AIN. LSIL includes what were previously classified as mild dysplasia, anal intraepithelial neoplasia I (AIN I), anal SIL (ASIL), and condyloma acuminatum.^103^ Anal condylomas should be thoroughly sampled as both LSIL and HSIL may be seen in anal condylomas due to infection with multiple HPV subtypes.^104^, ^105^, ^106^ HSIL includes lesions that were previously classified as moderate dysplasia, severe dysplasia, AIN II, AIN III, carcinoma in situ, Bowen's disease, and Bowenoid papulosis. p16 immunohistochemistry is characteristically diffusely positive and can distinguish HSIL from reactive changes.^103^ Although most AIN are related to HPV, the 6th edition recognises that subsets arise independently of HPV that are analogous to either *TP53*‐mutant or *TP53*‐wild type vulvar intraepithelial neoplasia.^107^, ^108^, ^109^

### Gall bladder

In the gall bladder, mass‐forming intraepithelial neoplasms are provisionally designated ICNs, which are further subcategorised as intracholecystic papillary (ICPN) and tubular (ICTN) neoplasms, in line with existing subtypes in the pancreas and the bile ducts. Pyloric gland adenoma is currently renamed as gastric subtype of ICPN.

### Bile ducts and pancreas: grading of precursors

All precursor lesions are now graded according to a two‐tiered classification system, low‐grade and high‐grade, with the former low‐grade and intermediate‐grade dysplasia now combined to be reclassified as low‐grade^110^; this is consistent with tumours in the tubular gastrointestinal tract.

### Intrahepatic cholangiocarcinoma

For this entity, the division into small duct and large duct intrahepatic cholangiocarcinomas, as proposed in the 5th edition^111^ has resulted in two separate entities: small duct (SD‐ICCA) and large duct intrahepatic cholangiocarcinoma (LD‐ICCA). This recognises fundamental differences between these two new entities with regard to aetiology, pathogenesis, epidemiology, macroscopy, histopathology, molecular alterations and finally clinical and therapeutic profile.

### NETs

The classification of neuroendocrine neoplasms has continued to evolve in the 6th edition with the intention that it will lay the foundation for greater uniformity in neuroendocrine neoplasm classification across all organ systems. It is emphasised that expression of at least two neuroendocrine markers (preferably including one of chromogranin A, synaptophysin, and INSM1) is required to confirm neuroendocrine differentiation, except in highly specific and unusual circumstances (e.g., the tuft cell variant of small cell carcinoma). CD56 is considered insufficiently specific to be used as a marker of neuroendocrine differentiation. The application of digital image analysis for Ki67 proliferative index determination is endorsed, provided that it has been validated against the gold standard (formal Ki67 evaluation of at least 500 neoplastic cells).^112^ If the mitotic count and Ki67 proliferative index potentially yield different grades, the higher grade (usually based on the Ki67 index) should be provided.

Greater guidance is given on the multiple parameters used to distinguish NETs from neuroendocrine carcinomas (NECs), noting that the distinction is entirely morphological and not based on any one feature, but rather a constellation of findings. It is acknowledged that NETs may evolve NEC‐like morphological features (diffuse growth, high‐grade cytological atypia, necrosis) over time, particularly after treatment, and rarely, this may be associated with the accumulation of NEC‐like molecular events (*RB1* and *TP53* somatic mutations).^113^, ^114^, ^115^ Although it is clear that these changes are associated with adverse outcomes, such events are rare and it is unclear whether these should be considered ‘*G3 NET with NEC‐like morphology*’ or ‘*NEC arising from NET*’.^113^

The fundamental approach to grading NETs has not changed significantly in the 6th edition, but in addition to recognition of ALCs, there are some nuances and evolving concepts summarised in Table 4. In many organ systems (e.g., pulmonary carcinoid/NET or medullary thyroid carcinomas, which are the NET of the thyroid), the presence of tumour necrosis is a validated part of the grading systems. There would be clear advantages in grading NETs of all organ systems using the same parameters. Although there is some evidence that tumour necrosis could form part of the grading schemes of some NETs of the gastrointestinal tract, with the best evidence being for pancreatic NETs,^116^ there is less evidence for other NETs, such as small bowel NETs. In an attempt to gather evidence for the potential future inclusion of necrosis in gastrointestinal tract NET grading, while not overstating the current evidence base, it was decided to record the presence or absence of tumour necrosis alongside the grade in all NETs. For example, G2 NET positive for tumour necrosis, or G3 NET negative for tumour necrosis. For the assessment of necrosis, only the presence of true tumour necrosis (not infarct‐like necrosis) is considered significant.

**Table 4:** Classification and grading of neuroendocrine neoplasms of the digestive system (6th edition)

Definition		Differentiation	Grading			Tumour necrosis
			Grade	Mitotic count^a^	Ki67 index^b^	
Pure NEN	NET	Well differentiated	G1	<2	<3	Present/absent
			G2	2–20	3–20	
			G3	>20	>20	
	NEC	Poorly differentiated	^c^	>20	>20	Not required^d^
Nonpure NEN	MiNEN	Variable	Variable			Present/absent^e^
	Amphicrine‐like carcinoma	Carcinoma	Variable			Not required

Amphicrine‐like carcinoma, amphicrine‐like adenocarcinoma or amphicrine‐like squamous cell carcinoma; G, grade; MiNEN, mixed neuroendocrine non‐neuroendocrine neoplasm; NEC, neuroendocrine carcinoma; NET, neuroendocrine tumour.

^a^ In areas of 2 mm^2^.

^b^ % of positive cells stained with Ki67 clone MIB‐1 in areas of highest nuclear labelling (hot spot); in the case of grade discrepancy between mitotic count and Ki67 the highest is to be indicated.

^c^ NECs are high‐grade by definition, therefore grade is not assessed further.

^d^ The presence of tumour necrosis is one factor, which may support the diagnosis of NEC over NET but is not definitive on its own and not mandatory when reporting NEC or amphicrine‐like carcinoma.

^e^ In MiNEN the presence of tumour necrosis need only be assessed in the neuroendocrine component if that component is a NET; in amphicrine‐like carcinoma the assessment of tumour necrosis is not required.

Studies of large cohorts of patients with pancreatic NETs (PanNETs) have provided good evidence that grade 2 tumours could potentially be subclassified into grade 2A with Ki67 of 3% to <10% and grade 2B with Ki67 of 10% to ≤20%.^113^, ^117^, ^118^, ^119^, ^120^, ^121^ There is insufficient data at this stage to confirm that subdivision of G2 NETs is applicable to tumours arising elsewhere in the gastrointestinal tract. Therefore, it was felt that it was appropriate to specifically acknowledge this potential subdivision in the pancreatic NET section without modifying the pre‐existing grading parameters for all NETs. Nevertheless, this illustrates the value of the Ki67 proliferative index as a continuous variable.

### Metastases: CUP site

CUP refers to a metastatic epithelial malignancy where the primary site of origin has not been identified despite comprehensive clinical, radiological and pathological investigation, including immunohistochemical and molecular testing; thus, CUP is regarded as an operational term. Now, it has been more specifically described compared with the 5th edition. CUP can be broadly divided into those where the clinical, radiological, immunohistochemical and molecular profile suggests origin from within or outside the gastrointestinal tract. These are further divided into favourable and unfavourable groups. The favourable group in general benefits from site‐specific therapy,^122^ whereas in the unfavourable group, the optimal therapy is not defined. The only favourable subtype of CUP with a gastrointestinal‐like profile is the colon‐like subtype (defined as CDX2 and CK20 positive, and CK7 negative, or showing a similar molecular profile to colorectal carcinoma).^123^, ^124^, ^125^, ^126^

## Conclusion

Here, we provide an overview of the most relevant changes, improvements and new entities in the new 6th edition of the WCT of the digestive system. Significant structural, molecular, and diagnostic updates are introduced to standardise terminology and improve clinical relevance. Epithelial tumours are organised by site, while neuroendocrine, mesenchymal and haematolymphoid tumours have dedicated chapters. Key refinements include revised grading systems, redefinition of undifferentiated carcinoma and unified approaches to dysplasia and precursor lesions. New entities include oesophageal epidermoid metaplasia, sonic hedgehog HCA, SDH‐deficient GIST, and ALC, amongst others. Anal canal neoplasia terminology aligns with HPV‐related classifications, and CUP is now covered, reflecting advances in immunohistochemical and molecular profiling relevant to precision oncology.

## Author contributions

MJA, IE, AJG, RHH, JK, MK, EAM, FA, FC, GC, GL, ADP, GR, MR, PS, AS, JY, JCH, JK, BR, MR‐M, ARS, CS, PP, HW, CG, BIIR, DL, and IDN contributed to writing the text, preparing figures and tables, and all reviewed and agreed on the final manuscript.

## Funding information

None.

## Conflict of interests

The authors declare no competing interests. The content of this article represents the personal views of the authors and does not represent the views of the authors' employers and associated institutions. When authors are identified as personnel of the International Agency for Research on Cancer/World Health Organization, the authors alone are responsible for the views expressed in this article and they do not necessarily represent the decisions, policy, or views of the International Agency for Research on Cancer/World Health Organization.

## Data Availability

No primary new data was used in this review article manuscript. The manuscript refers to the WHO Classification of Tumours of the Digestive System, 6th edition blue book (International Agency for Research on Cancer, Lyon, France), scheduled to be published in the Spring of 2026.
